# Genome-wide identification and expression analysis of the calmodulin-binding transcription activator (*CAMTA*) gene family in wheat (*Triticum aestivum* L.)

**DOI:** 10.1186/s12863-020-00916-5

**Published:** 2020-09-14

**Authors:** Fan Yang, Fu-shuang Dong, Fang-hui Hu, Yong-wei Liu, Jian-fang Chai, He Zhao, Meng-yu Lv, Shuo Zhou

**Affiliations:** 1 Institute of Genetics and Physiology, Hebei Academy of Agriculture and Forestry Sciences/Plant Genetic Engineering Center of Hebei Province, Shijiazhuang, 050051 People’s Republic of China; 2 Agriculture and Rural Bureau of Nanhe County, Xingtai, 054400 People’s Republic of China

**Keywords:** CAMTA, Wheat, Genome-wide identification, Gene expression

## Abstract

**Background:**

Plant calmodulin-binding transcription activator (CAMTA) proteins play important roles in hormone signal transduction, developmental regulation, and environmental stress tolerance. However, in wheat, the *CAMTA* gene family has not been systematically characterized.

**Results:**

In this work, 15 wheat *CAMTA* genes were identified using a genome-wide search method. Their chromosome location, physicochemical properties, subcellular localization, gene structure, protein domain, and promoter *cis*-elements were systematically analyzed. Phylogenetic analysis classified the *TaCAMTA* genes into three groups (groups A, B, and C), numbered 7, 6, and 2, respectively. The results showed that most *TaCAMTA* genes contained stress-related *cis*-elements. Finally, to obtain tissue-specific and stress-responsive candidates, the expression profiles of the *TaCAMTAs* in various tissues and under biotic and abiotic stresses were investigated. Tissue-specific expression analysis showed that all of the 15 *TaCAMTA* genes were expressed in multiple tissues with different expression levels, as well as under abiotic stress, the expressions of each *TaCAMTA* gene could respond to at least one abiotic stress. It also found that 584 genes in wheat genome were predicted to be potential target genes by CAMTA, demonstrating that CAMTA can be widely involved in plant development and growth, as well as coping with stresses.

**Conclusions:**

This work systematically identified the *CAMTA* gene family in wheat at the whole-genome-wide level, providing important candidates for further functional analysis in developmental regulation and the stress response in wheat.

## Background

Ca^2+^ signals, one of the most important secondary messengers in plants, are widely involved in many adaptive and developmental processes [1]. In plants, there are three main classes of Ca^2+^ sensors to decode and transmit the Ca^2+^ signals, including calmodulin (together with calmodulin-like proteins) (CaMs/CMLs), calcium-dependent protein kinases (CDPKs), and calcineurin B-like proteins (CBLs) [2]. Most of the calmodulin/calmodulin-like proteins execute their biological functions by binding to calmodulin-binding proteins (CaMBPs), including transcription factors, protein kinases, ion channels, and enzymes, with the exception of CaM7, which can act as a transcription factor to directly regulate the expression of the *HY5* gene [3]. To our knowledge, plant CaMs can regulate at least 90 transcription factors, including calmodulin-binding transcription activators (CAMTAs) [4].

CAMTA proteins are characterized by several conserved domains, including a unique DNA-binding domain (CG-1), a transcription factor immunoglobulin-like DNA-binding domain (TIG), ankyrin repeats (ANK), IQ motifs (IQXXXRGXXXR), and a Ca^2+^-dependent calmodulin-binding domain (CaMBD) [5–7]. To date, the *CAMTA* gene family has been identified in a wide variety of plant species, such as *Arabidopsis* (*Arabidopsis thaliana*, six members) [7], tomato (*Solanum lycopersicum*, seven members) [8], rice (*Oryza sativa*, seven members) [9], grape (*Vitis vinifera*, 10 members) [10], maize (*Zea mays*, nine members) [11], soybean (*Glycine max*, 15 members) [12], rape (*Brassica napus*, 18 members) [13], alfalfa (*Medicago sativa*, seven members) [14], poplar (*Populus trichocarpa*, seven members) [4], and citrus (*Citrus sinensis and Citrus clementina*, nine members) [15].

CAMTAs have been shown to be extensively involved in plant growth and developmental regulation, as well as in biotic and abiotic stress tolerance. In *Arabidopsis*, CAMTA1 and CAMTA2 work in concert with CAMTA3 to directly bind to the promoter of C-repeat binding factor2 (*CBF2*) to induce expression, leading to increased plant freezing tolerance [16, 17]. While AtCAMTA1 also positively regulates drought responses by regulating a few stress-responsive genes, including responsive to dehydration26 (*RD26*), early response to dehydration7 (*ERD7*), responsive to ABA18 (*RAB18*), lipid transfer proteins (*LTPs*), cold-regulated78 (*COR78*), *CBF1*, and heat shock proteins (*HSPs*) [18], AtCAMTA3 can act as a negative regulator of plant immunity to modulate pathogen defense responses by activating the EDS1-mediated salicylic acid (SA) signaling [19]. A recent study showed that *TaCAMTA4* may function as a negative regulator of the defense response against *Puccinia triticina*, since the virus-induced gene silencing (VIGS)-based knockdown of *TaCAMTA4* resulted in the enhanced resistance to *P. triticina* race 165 [20]. This suggested that one CAMTA member usually participates in multiple signaling pathways, while multiple CAMTA members often work together to participate in one signaling pathway.

Here, we obtained 15 *TaCAMTA* genes from wheat genomes. Their chromosome location, physicochemical properties, subcellular localization, gene structure, protein domain, promoter *cis*-elements, and expression profiles in multiple tissues as well as in response to stresses were systematically analyzed. Our work has established a foundation for the further analysis of wheat *CAMTA* genes and provides a basic understanding of their roles in development and stress responses.

## Results and discussion

### Identification of the *TaCAMTA* gene family in wheat

**Fig. 1:**
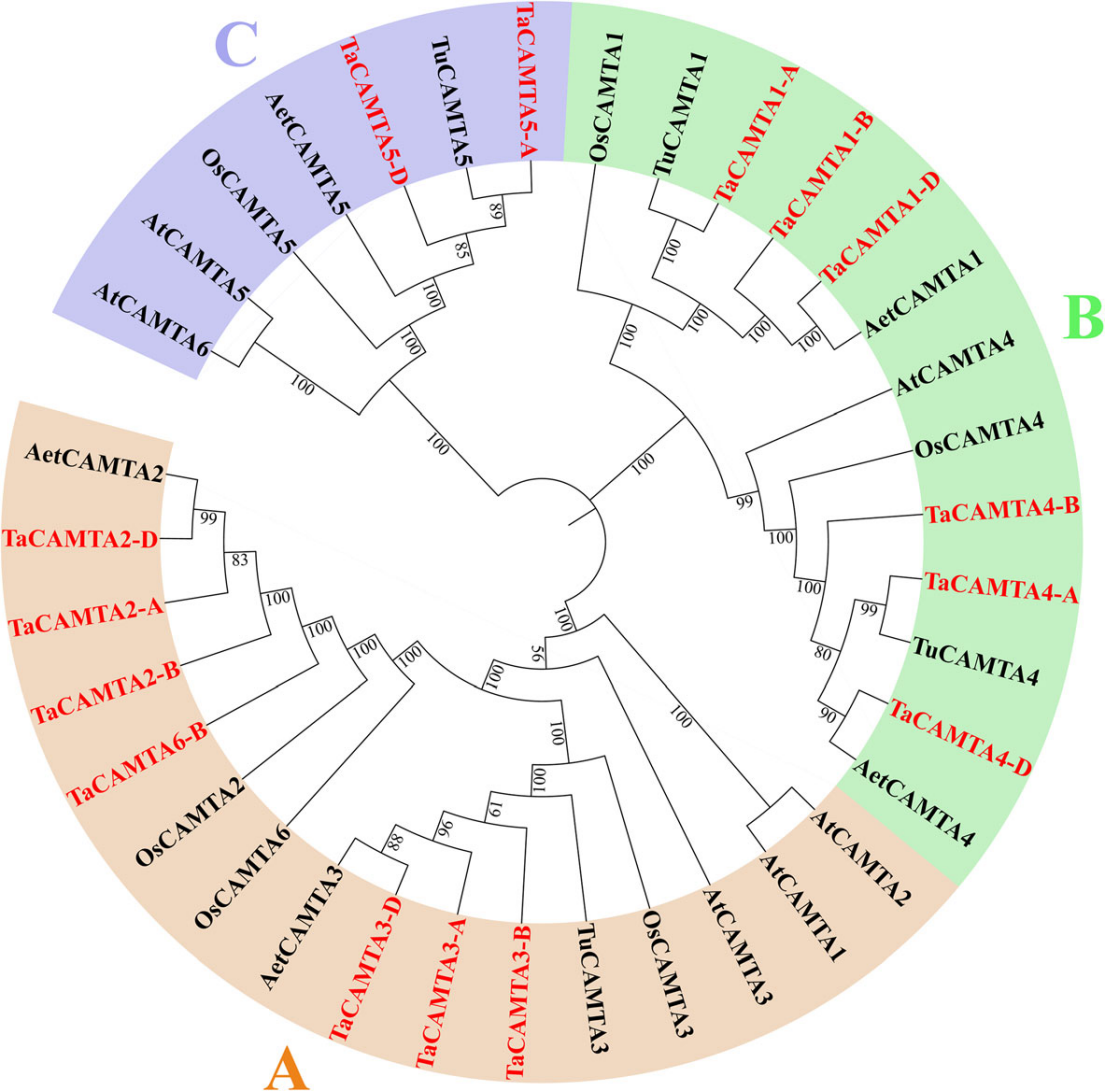
Phylogenetic relationships of the CAMTA homologs in different species

Using the method described below, a total of 15 *TaCAMTA* genes were identified in wheat. Since the *TaCAMTA* genes were clustered into six homoeologous groups, these genes were designated as *TaCAMTA1* to *TaCAMTA6* according to their homology with rice *CAMTA* genes, plus a suffix corresponding to the specific wheat genome identifier (A, B, or D) for each gene name (Table 1, Fig. 1). For example, the *TaCAMTA1* genes in genomes A, B, and D were named *TaCAMTA1-A*, *TaCAMTA1-B*, and *TaCAMTA1-D*, respectively. The results showed that *TaCAMTA1*, *2*, *3*, and *4* contained three homolog genes (*TaCAMTA1-A/B/D*, *2-A/B/D*, *3-A/B/D*, and *4-A/B/D*), while *TaCAMTA5* harbored two (*TaCAMTA5-A/D*), and *TaCAMTA6* possessed one (*TaCAMTA6-B*). The highest number (eight genes: *TaCAMTA3-A/B/D*, *4-A/B/D*, and *5-A/D*) of *TaCAMTA* genes was found in homoeologous group 2, three *TaCAMTA* genes (*TaCAMTA1-A/B/D*) in homoeologous group 3, three *TaCAMTA* genes (*TaCAMTA2-A/B/D*) in homoeologous group 4, and one *TaCAMTA* gene (*TaCAMTA6-B*) in homoeologous group 5, while no *TaCAMTA* gene was identified in homoeologous groups 1, 6, and 7. Information relating to the 15 *TaCAMTA* genes, including gene names, locus IDs, open reading frame (ORF) lengths, chromosome locations, and the deduced polypeptides is provided in Table 1. The predicted TaCAMTA proteins contain 805 (TaCAMTA1-B) to 1067 (TaCAMTA2-B) amino acid residues, with molecular weights ranging from 90.82 kDa (TaCAMTA1-B) to 119.32 kDa (TaCAMTA2-A), and the isoelectric points ranged from 5.14 (TaCAMTA4-B) to 8.96 (TaCAMTA5-A) (Table 1).

**Table 1:** Information of the 15 *CAMTA* gene members in wheat

Gene	Locus ID	Chr. location^**a**^	ORF length (bp)	Length (AA)	MW (kDa)	pI	Subcellular localization	Ortholgous genes in rice
*TaCAMTA1-A*	TraesCS3A02G433300	3A(−):674751244–674,757,428	3420	821	92.48	7.5232	Nucleus	*OsCAMTA1*
*TaCAMTA1-B*	TraesCS3B02G469100	3B(−):715886740–715,893,072	3253	805	90.82	7.1271	Nucleus	
*TaCAMTA1-D*	TraesCS3D02G426700	3D(−):539903862–539,910,357	3499	818	92.51	7.2482	Nucleus	
*TaCAMTA2-A*	TraesCS4A02G407100	4A(−):679981642–679,990,156	3806	1066	119.32	5.8827	Nucleus	*OsCAMTA2*
*TaCAMTA2-B*	TraesCS4B02G306300	4B(−):595277477–595,286,089	3764	1067	119.19	5.9095	Nucleus	
*TaCAMTA2-D*	TraesCS4D02G304500	4D(−):472932312–472,941,309	3988	1066	119.16	5.8827	Nucleus	
*TaCAMTA3-A*	TraesCS2A02G163000	2A(+):115413507–115,422,090	3443	1026	113.84	5.7467	Nucleus	*OsCAMTA3*
*TaCAMTA3-B*	TraesCS2B02G188800	2B(+):164418775–164,427,330	3780	1027	114.11	5.8397	Nucleus	
*TaCAMTA3-D*	TraesCS2D02G169900	2D(+):113911362–113,919,591	3783	1026	113.90	5.7476	Nucleus	
*TaCAMTA4-A*	TraesCS2A02G283800	2A(+):475870112–475,878,163	3530	1027	114.42	5.2722	Nucleus	*OsCAMTA4*
*TaCAMTA4-B*	TraesCS2B02G300800	2B(+):423658566–423,666,571	3407	1028	114.50	5.1358	Nucleus	
*TaCAMTA4-D*	TraesCS2D02G282800	2D(+):355676387–355,685,067	3899	1030	114.91	5.182	Nucleus	
*TaCAMTA5-A*	TraesCS2A02G229400	2A(−):258345594–258,361,280	3075	907	101.83	8.9645	Nucleus	*OsCAMTA5*
*TaCAMTA5-D*	TraesCS2D02G237300	2D(−):239973233–239,987,968	2841	907	101.94	8.8824	Nucleus	
*TaCAMTA6-B*	TraesCS5B02G521100	5B(−):683109213–683,139,863	3240	891	99.46	7.0588	Nucleus	*OsCAMTA6*

*ID* identity, *Chr* chromosome, *ORF* open reading frame, *AA* amino acids, *pI* isoelectric point, *MW* molecular weight

a Chromosomal location: “+” and “−” indicate the forward and reverse strand, respectively

The size of the *CAMTA* gene family in wheat is similar to that of oilseed rape (*B. napus*) and soybean (*G. max*) with 18 and 15 members [12, 13], respectively, but is higher than that of *A. thaliana* with six members, citrus (*C. sinensis* and *C. clementina*) with nine members, maize (*Z. mays*) with nine members, and alfalfa (*M. truncatula*) with seven members [5, 11, 14, 15]. The higher number of *CAMTA* genes may be due to gene duplication during chromosome polyploidization, since oilseed rape and soybean are tetraploid, whereas wheat is allohexaploid (AABBDD).

The subcellular locations were predicted with Plant-mPLoc. According to the results, all 15 wheat CAMTA proteins were located in the nucleus, which corroborates recent studies where the CAMTA proteins have typically been located in the nucleus [4, 21], confirming that their main function is to regulate the expression of other genes as transcription factors.

### Phylogenetic analysis of the TaCAMTAs

To investigate the phylogenetic relationships of the *CAMTA* gene families, a phylogenetic tree of *CAMTAs* from five species, including wheat, *Triticum urartu*, *Aegilops tauschii*, *A. thaliana*, and rice, was constructed using the neighbor-joining (NJ) algorithm. The *CAMTA* gene families were highly conserved during the evolution of these species (Fig. 1). All of the 36 proteins from the five species were distinctly clustered into three groups (groups A, B, and C). Seven wheat *CAMTAs* (*TaCAMTA2-A/−B/−D*, *3-A/B/D*, and *6-B*), one *T. urartu CAMTA* (*TuCAMTA3*), two *Ae. tauschii CAMTAs* (*AetCAMTA2*, and *3*), three rice *CAMTAs* (*OsCAMTA2*, *3*, and *6*), and three *Arabidopsis CAMTAs* (*AtCAMTA1*, *2*, and *3*) were clustered into group A. In addition, six wheat *CAMTAs* (*TaCAMTA1-A/−B/−D*, and *TaCAMTA4-A/−B/−D*), two *T. urartu CAMTAs* (*TuCAMTA1*, and *4*), two *Ae. tauschii CAMTAs* (*AetCAMTA1* and *4*), two rice *CAMTAs* (*OsCAMTA1*, and *4*), and one *Arabidopsis CAMTA* (*AtCAMTA4*) grouped into group B, while two wheat *CAMTAs* (*TaCAMTA5-A/−D*), one *T. urartu CAMTA* (*TuCAMTA5*), one *Ae. tauschii CAMTA* (*AetCAMTA5*), one rice *CAMTA* (*OsCAMTA5*), and two *Arabidopsis CAMTAs* (*AtCAMTA5* and *6*) clustered into group C.

An unrooted phylogenetic tree was constructed using MEGA-X with the NJ algorithm and 1000 bootstrap replicates. The bootstrap values are displayed next to the branches, and the wheat *CAMTAs* are marked in red. The *CAMTA* gene ID numbers are listed as follows: *A. thaliana*: *AtCAMTA1* (AT5G09410), *AtCAMTA2* (AT5G64220), *AtCAMTA3* (AT2G22300), *AtCAMTA4* (AT1G67310), *AtCAMTA5* (AT4G16150), *AtCAMTA6* (AT3G16940); rice: *OsCAMTA1* (LOC_Os01g69910), *OsCAMTA2* (LOC_Os03g09100), *OsCAMTA3* (LOC_Os07g43030), *OsCAMTA4* (LOC_Os04g31900), *OsCAMTA5* (LOC_Os07g30774), *OsCAMTA6* (LOC_Os10g22950); *T. urartu*: *TuCAMTA1* (TRIUR3_22499-P1), *TuCAMTA3* (TRIUR3_23792-P1), *TuCAMTA4* (TRIUR3_26386-P1), *TuCAMTA5* (TRIUR3_19786-P1); *Ae. tauschii*: *AetCAMTA1* (XP_020189402), *AetCAMTA1* (XP_020179695), *AetCAMTA1* (XP_020196708), *AetCAMTA1* (XP_020147564), and *AetCAMTA1* (XP_020186933).

### Gene architectures and protein domain structures of the *TaCAMTA* members

**Fig. 2:**
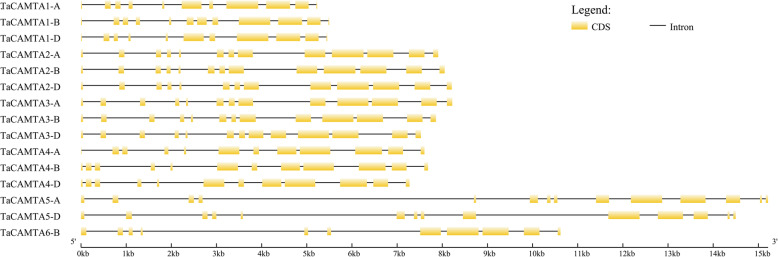
Gene architectures of the *TaCAMTA* genes

The number of introns in all of the 15 *TaCAMTA* genes varied from 10 to 13, in which three *CAMTA* genes (*TaCAMTA1-A/D* and *6-B*) possessed 10 introns, four *CAMTA* genes (*TaCAMTA1-B* and *4-A/B/D*) possessed 11 introns, six *CAMTA* genes (*TaCAMTA2-A/B/D* and *3-A/B/D*) possessed 12 introns, and two *CAMTA* genes (*TaCAMTA5-A/D*) possessed 13 introns (Fig. 2). Similar genomic structures of the CAMTA genes have been observed in other plant species, suggesting the conservation of CAMTA genes across plant species [8, 11, 12, 21].

The exon-intron structures of the *TaCAMTA* genes were analyzed by comparing the coding sequences and the corresponding genomic sequences using the Gene Structure Display Server (GSDS, http://gsds.cbi.pku.edu.cn/). The black box indicates exons, and the black line indicates introns.

**Fig. 3:**
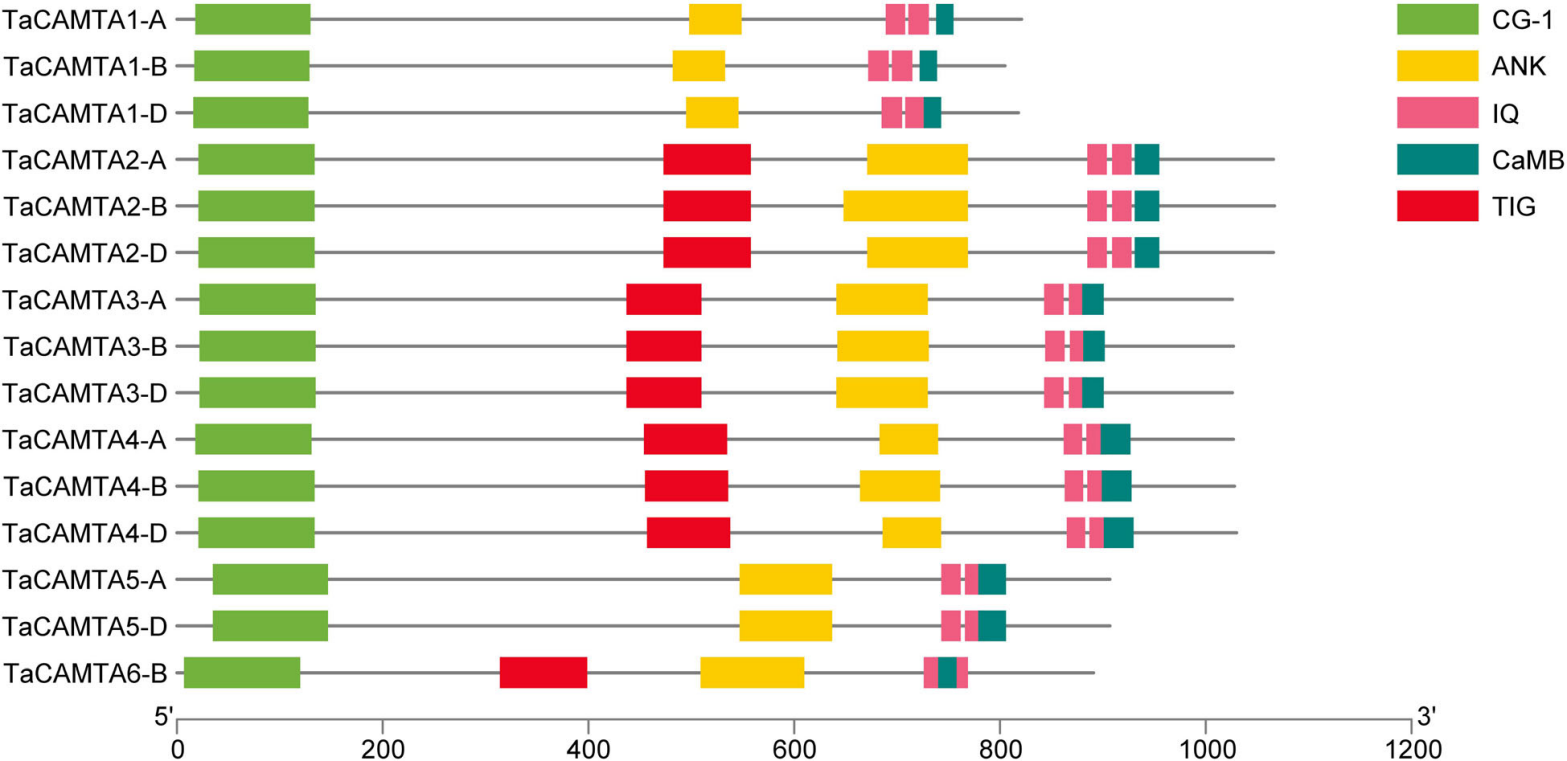
Protein domain structure of the TaCAMTAs

Ten TaCAMTA proteins (TaCAMTA2-A/B/D, 3-A/B/D, 4-A/B/D, and 6-B) were predicted to contain all of the conserved domains of a typical CAMTA protein, including a CG-1 DNA-binding domain (Pfam03859), a TIG domain involved in non-specific DNA binding (Pfam01833), several ankyrin repeats (Pfam12796), an IQ motif (Pfam00612), and a calmodulin-binding domain (CaMB) (Fig. 3). Additionally, five TaCAMTA proteins (TaCAMTA1-A/B/D and TaCAMTA5-A/D) contained all of the conserved domains except for the TIG domain, which is consistent with previous studies that CAMTAs can be divided into two groups based on whether the TIG domain is present [22].

It has been confirmed that the IQ motif is able to bind with CaM in a Ca^2+^-independent manner, while the CaMB domain interacts with CaM in a Ca^2+^-dependent way [5, 7, 8]. It is interesting to note that all the wheat CAMTAs contain the IQ motif and a CaMB domain, indicating that wheat CAMTAs may interact with CaM in both a Ca^2+^-dependent and Ca^2+^-independent manner.

Analysis of the functionally conserved domains was performed using the Pfam database (http://pfam.janelia.org/) and NCBI Conserved Domains Search online tool (https://www.ncbi.nlm.nih.gov/Structure/cdd/wrpsb.cgi). CaM-binding domains (CaMBD) were analyzed in the Calmodulin Target Database (http://calcium.uhnres.utoronto.ca/ctdb/ctdb/). The domain structures of the TaCAMTAs were illustrated using TBtools software. CG-1, CG-1 DNA binding domain; TIG, TIG domain involved in non-specific DNA binding, ANK, ankyrin repeats responsible for mediating protein-protein interactions; IQ, Ca^2+^-independent CaM-binding IQ motifs; CaMBD, Ca^2+^-dependent CaM binding domain.

### *Cis*-acting regulatory elements in the promoters of the *TaCAMTAs*

Several stresses/stimuli response *cis*-acting elements in the promoter regions (2000 bp upstream of the translation start site ATG) of the 15 *TaCAMTA* genes were predicted. Seven *cis*-elements were used in this study: abscisic acid (ABA)-responsive element (ABRE: ACGTG, ACGTSSSC, or MACGYGB) [23], SA-responsive promoter element (SARE: TGACG) [24], environmental signal response element (G-box: CACGTG) [25], WRKY binding site (W-box: TTGAC, or TGACC/T) [26, 27], phosphate starvation-responsive element (P1BS: GNATATNC) [28], sulfur-responsive element (SURE: GAGAC) [29], and the CAMTA binding site (CG-box: A/C/GCGCGG/T/C) [5].

The results showed that there were various known stresses/stimuli-related *cis*-acting elements that existed in the promoter regions of the 15 *TaCAMTA* genes. ABRE, SARE, W-box, and CG-box could be found in the promoter of all the 15 *TaCAMTA* genes, and four *TaCAMTAs* (*TaCAMTA1-D*, *3-B*, *4-A*, and *4-D*) contained all seven types of *cis*-elements in the promoter region, including ABRE, SARE, G-box, W-box, P1BS, SURE, and CG-box. Meanwhile, the remainder of the 11 *TaCAMTA* genes contained at least five *cis*-elements in their promoter region (Table 2). It has been reported that more stress-related *cis*-elements are located in the promoter regions of wheat *CAMTA* genes than other plant species [13, 14], indicating that wheat *CAMTA* genes may be more widely involved in the plant response to stress.

**Table 2:** Numbers of stress-related *cis*-elements in the promoter regions of the *TaCAMTA* genes

	ABRE	SARE	G-box	W-box	P1BS	SURE	CG-box
*TaCAMTA1-A*	11	1	1	4	1	0	17
*TaCAMTA1-B*	4	3	0	5	1	1	11
*TaCAMTA1-D*	7	7	2	3	1	3	10
*TaCAMTA2-A*	3	3	1	9	0	3	4
*TaCAMTA2-B*	10	1	1	5	0	3	14
*TaCAMTA2-D*	6	2	1	9	0	3	8
*TaCAMTA3-A*	1	2	0	5	0	2	3
*TaCAMTA3-B*	4	5	1	1	1	4	6
*TaCAMTA3-D*	2	5	1	2	0	2	5
*TaCAMTA4-A*	9	2	1	8	1	5	9
*TaCAMTA4-B*	5	4	2	7	0	3	10
*TaCAMTA4-D*	8	4	1	6	1	4	10
*TaCAMTA5-A*	1	1	0	7	0	3	5
*TaCAMTA5-D*	1	3	1	6	0	1	3
*TaCAMTA6-B*	1	1	0	11	1	2	2

*ABRE* ABA-responsive element, *SARE* SA-responsive promoter element, *G-box* environmental signal response element, *W-box* WRKY binding site, *P1BS* phosphate starvation-responsive element, *SURE* sulfur-responsive element, *CG-box* the CAMTA binding site

### Tissue-specific expression patterns of the *TaCAMTA* genes

**Fig. 4:**
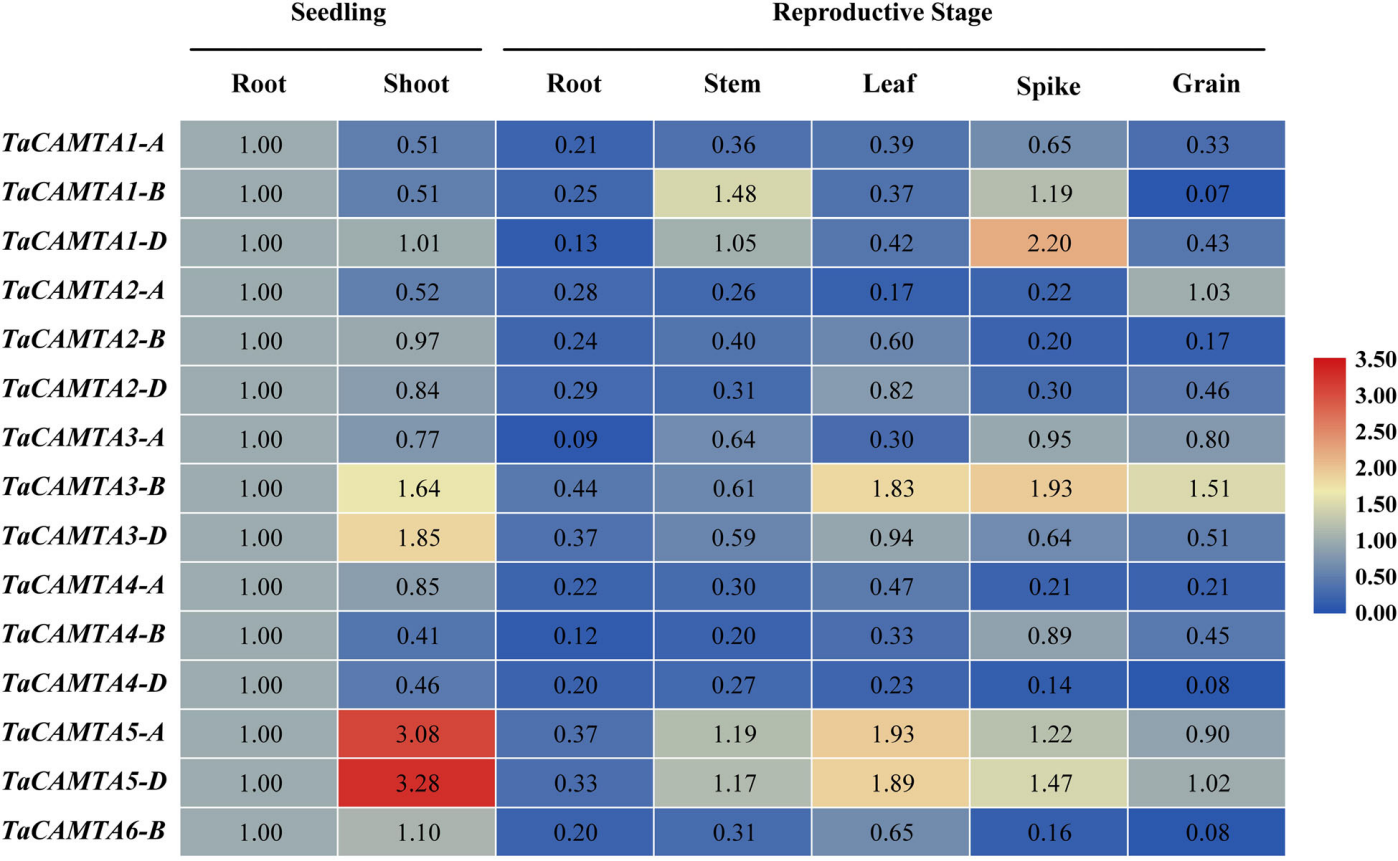
Expression patterns of the *TaCAMTA* genes in multiple tissues

To elucidate the possible functions of the *TaCAMTA* genes in wheat, qRT-PCR assay was performed to investigate the spatial expression patterns of the *TaCAMTAs*. The results showed that all of the 15 *TaCAMTA* genes were expressed in multiple tissues with different expression levels. *TaCAMTA3-D*, *5-A*, and *5-D* showed highest expression level in shoot during seedling stage, while highest expression level of *TaCAMTA1-D* and *3-B* was observed in spike during reproductive stage, suggesting that various *CAMTA* gene members maintain different functions in wheat growth and development (Fig. 4).

Expression of *TaCAMTAs* were analyzed by qRT-PCR in root and shoot of ten-day-old seedlings, root, stem, leaf, spike at flowering in reproductive stage, and grain 15 DAA (days after athesis). The relative expression levels were normalized to 1 in roots of ten-day-old seedlings (0 h).

### Expression profiles of the *TaCAMTA* genes during abiotic stress

**Fig. 5:**
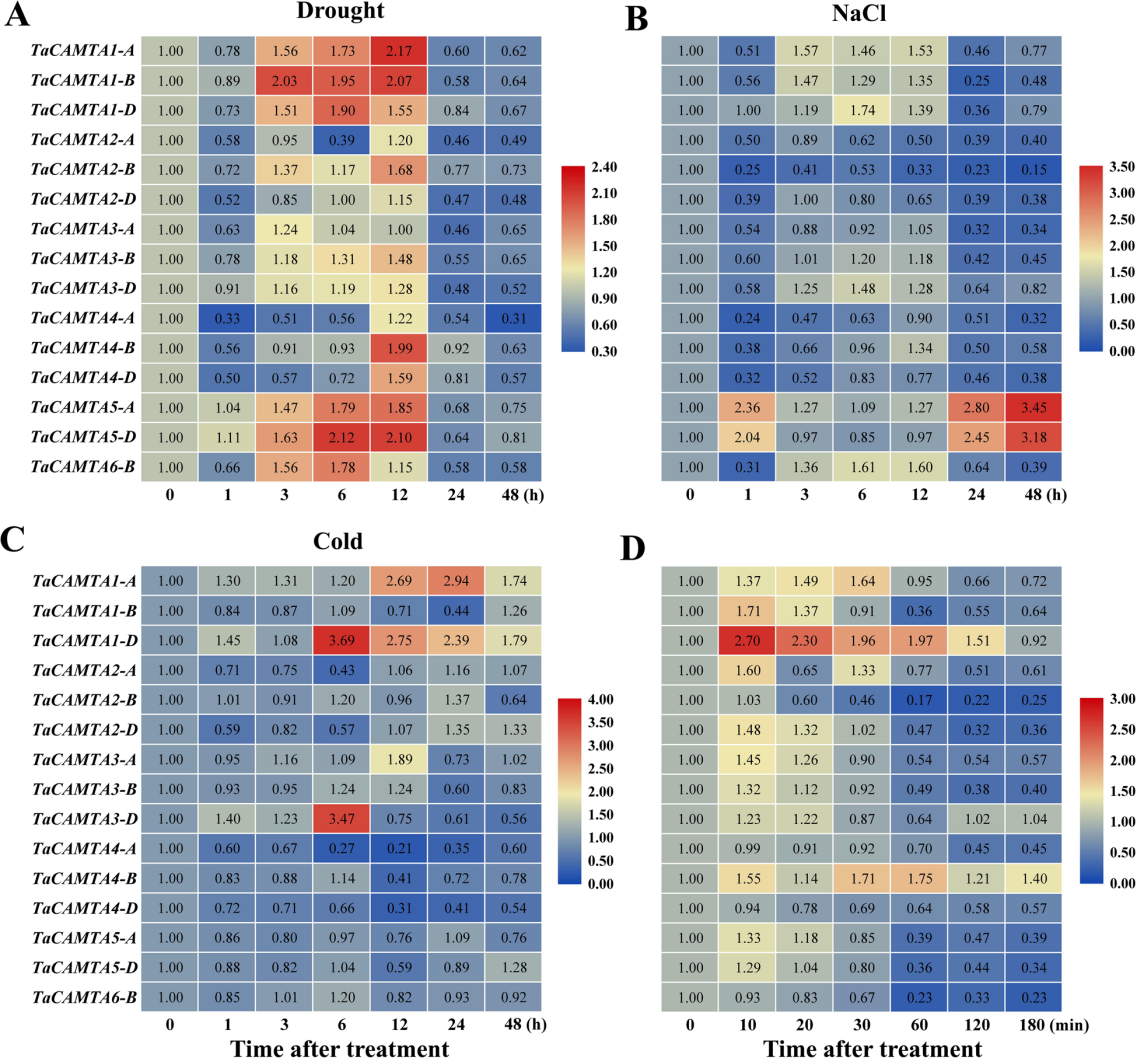
Expression profiling of the *TaCAMTA* genes under abiotic stress

Previous studies have shown that plant CAMTAs could be involved in diverse environmental stresses. *AtCAMTA1* and *SlSR1L* played a positive function in drought stress in *Arabidopsis* and tomato [18, 30], while plant *CAMTAs* also respond to salt and cold stress [11, 16, 31]. However, to date there is no information available on wheat *CAMTAs* involved in abiotic stresses. In this light, the expression profiles of the *TaCAMTAs* were analyzed under drought, NaCl, cold and heat stress. Under drought stress, *TaCAMTA1-A*, *1-B*, *1-D*, *2-B*, *4-B*, *4-D*, *5-A*, *5-D* and *6-B* were significantly up-regulated, while the expressions of *TaCAMTA2-A*, *2-D*, *3-A*, *3-D* were moderately down-regulated (Fig. 5a). In response to NaCl stress, the expressions of *TaCAMTA1-A*, *1-D*, *5-A*, *5-D* and *6-B* were enhanced, while the expressions of TaCAMTA2-A, 2-B, 2-D, 3-A, 3-B, 4-A, 4-B, 4-D were inhibited (Fig. 5b). In the cold treatment assay, the expressions of *TaCAMTA1-A*, *1-D*, *3-A*, and *3-D* increased dramatically, while the expressions of *TaCAMTA2-A*, *4-A*, *4-B*, and *4-D* decreased (Fig. 5c). In the heat treatment group, the expressions of *TaCAMTA1-A*, *1-B*, *1-D*, *2-A*, and *4-B* remarkably increased within one hour; by contrast, the expressions of *TaCAMTA2-B*, *2-D*, *3-B*, *4-A*, *5-A*, *5-D*, and 6*-B* were repressed, especially in the late stage of heat treatment (Fig. 5d).

It can been found that the expression of each *TaCAMTA* gene could respond to at least one abiotic stress, and *TaCAMTA1-A* and *1-D* could be up-regulated by all abiotic stresses used in this study, including drought, NaCl, cold and heat stress (Fig. 5), implying different regulations and functions of *TaCAMTA* gene members while coping with various abiotic stresses in wheat. It can also been found that the *CAMTA* genes from same homoeologous group showed similar expression patterns, such as *TaCAMTA1-A/B/D* under drought treatment (Fig. 5a), *TaCAMTA5-A/D* under NaCl treatment (Fig. 5b), and *TaCAMTA1-A/B/D* under heat shock stress (Fig. 5d). However, several homoeologous *CAMTA* genes from same group showed different expression patterns under stresses. For example, *TaCAMTA1-A/D* and *TaCAMTA3-A/D* were up-regulated by cold treatment, while the expressions of *TaCAMTA1-B* and *TaCAMTA3-B* were relatively stable (Fig. 5c). These results suggest that the homoeologous *CAMTA* genes from the same group generally have the same regulations and functions, while functional differentiation may have occurred in some homoeologous *CAMTA* genes.

Expression of *TaCAMTAs* were analyzed by qRT-PCR in roots of ten-day-old seedlings, which had been treated with 16.1% PEG 6000 (drought), 200 mM NaCl, 4 °C (cold) and 40 °C (heat) for indicated durations. The relative expression levels were normalized to 1 in unstressed plants (0 h).

### Prediction of target genes by CAMTA

It has been found that CAMTA has the specific binding activity to CGCG box in promoter of target genes [5]. In this study, a search of the data base revealed that *cis*-acting elements ACGCGG/CCGCGT were present in the promoter regions of about 584 genes (more than two copies) in wheat genome, which were considered as potential target genes by CAMTA (Additional file 1:Table S1). These genes are related to RNA regulation (69 genes), protein degradation (42 genes), signalling transduction (30 genes), biotic and abiotic stresses (17 genes), hormone metabolism (17 genes), and lipid metabolism (13 genes), demonstrating that CAMTA can be widely involved in plant development and growth, as well as coping with stresses.

## Conclusions

In conclusion, 15 *CAMTA* genes were identified in wheat in the present study. Analysis of the gene structure and protein domain, physicochemical properties, and the phylogenetic relationships indicated that the *CAMTA* gene family was highly conserved during plant evolution. Tissue-specific expression analysis showed that all of the 15 *TaCAMTA* genes were expressed in multiple tissues with different expression levels, suggesting that various *CAMTA* gene members maintain different functions in wheat growth and development. Under abiotic stress, the expressions of all the *TaCAMTA* genes could respond to at least one abiotic stress, implying different regulations and functions of *TaCAMTA* gene members while coping with various abiotic stresses in wheat. 584 genes in wheat genome were predicted to be potential target genes by CAMTA, demonstrating that CAMTA can be widely involved in plant development and growth, as well as coping with stresses. Our findings provide new insight into the *CAMTA* gene family in wheat as well as a foundation for further studies on the roles of *TaCAMTA* genes in wheat development and growth as well as the stress response.

## Methods

### Genome-wide identification of the *CAMTA* gene family

Protein sequences of *Triticum aestivum* (IWGSC1.1), *Triticum urartu* (ASM34745v1), and *Aegilops tauschii* (ASM34733v1) were obtained from the Ensemble plant database (http://plants.ensembl.org) to predict the *CAMTA* genes [32]. The Hidden Markov Model (HMM) profile of the CG-1 domain (PF03859), the ANK repeat domain (PF00023), and the IQ domain (PF00612) sequences were downloaded from the PFAM database [33] and used to examine all wheat protein sequences using the HMMER search tool with E-value <= 0.0001 [34]. The obtained protein sequences were checked using the National Center for Biotechnology Information (NCBI) - Conserved domain database (CDD) search (https://www.ncbi.nlm.nih.gov/cdd) to identify the conserved protein domain with the default parameters. The redundant sequences containing complete CG-1, ANK repeats, and the IQ domain were further removed by alignment, and the remainder were considered as putative *CAMTA* genes. Finally, the biochemical parameters of the TaCAMTA proteins were calculated using the Compute pI/MW tool in the ExPASy database with the default parameters (https://web.expasy.org/compute_pi/). Subcellular localization of the TaCAMTA proteins was predicted online by Plant-mPLoc with the default parameters (http://www.csbio.sjtu.edu.cn/bioinf/plant-multi/) [35].

### Phylogenetic tree construction and sequence analysis

Protein sequences from *Arabidopsis* and rice were obtained from NCBI (http://www.ncbi.nlm.nih.gov/) and Ensembl Plants (http://plants.ensembl.org/index.html). The amino acid sequences of all CAMTAs were aligned using the ClustalX program with the default parameters, and a phylogenetic tree was constructed in MEGA-X using the neighbor-joining method with 1000 bootstrap replicates [36]. The display of the phylogenetic tree was optimized using the Interactive Tree Of Life (iTOL) v4 [37].

The schematic structures of the *TaCAMTA* genes were analyzed online using the Gene Structure Display Server 2.0 based on exon/intron data (GSDS 2.0, http://gsds.cbi.pku.edu.cn/) [38]. The domain structures of the TaCAMTA proteins were analyzed in the Pfam database (http://pfam.janelia.org/) and NCBI Conserved Domains Search online tool against database CDD v3.18 with E-value threshold <= 0.01 (https://www.ncbi.nlm.nih.gov/Structure/cdd/wrpsb.cgi) [39]. The CaMB domain was specifically analyzed using Calmodulin Binding Site Search in the Calmodulin Target Database (http://calcium.uhnres.utoronto.ca/ctdb/ctdb/).

### Prediction of *cis*-acting elements in the *TaCAMTA* genes

To investigate the *cis*-elements in the promoter sequences of the *TaCAMTA* genes, 2-kb sequences upstream of the initiation codon (ATG) were collected from the Ensembl plant database (http://plants.ensembl.org) and subjected to the PLACE database with the default parameters (https://www.dna.affrc.go.jp/PLACE) [32, 40].

### Plant materials and treatments

Jinhe 9123, a wheat variety cultivated by our lab, was used in this study. For stress treatment experiments, seeds were surface-sterilized in 1% NaOCl and repeatedly rinsed with tap water, then seeded in 1/2 Hoagland nutrient solution after immersion and imbibition for 12 h [41], and hydroponically cultivated in the incubator with a 16/8-h photoperiod at 25 °C. Four ten-day-old homogeneous seedlings groups, each of which included 30 seedlings from three biological replicates, were subjected to different treatments, including 16.1% PEG6000, 200 mM NaCl and cold (4 °C) for 1, 3, 6, 12, 24 and 48 h, and heat (40 °C) for 10, 20, 30, 60, 120, 180 min. The shoot tissue was sampled for the experiment. For analyses of tissue-specific expression patterns, root and shoot from ten-day-old seedlings, root, stem, leaf, spike at flowering in reproductive stage, and grain 15 DAA were collected from wheat plants. Collected samples were immediately frozen in liquid nitrogen and stored at − 80 °C for RNA extraction.

### RNA isolation and gene expression analysis

Total RNA of collected samples was isolated using the EasyPure® Plant RNA Kit (ER301–01, Transgen, China). For reverse transcription, the first-strand cDNA was synthesized using a PrimeScript™ RT reagent Kit (RR047A, TaKaRa, Japan). Quantitative real-time PCR (qRT-PCR) for examination of the *TaCAMTAs* expression patterns were performed using the TB Green™ Premix Ex Taq™ II (RR820A, TaKaRa, Japan) with 7500 Real-Time PCR System (Applied Biosystem, USA). Gene specific and internal reference gene *TaActin* primers were listed in Additional file 2: Table S2. The qRT-PCR program was carried out as follows: predenaturation at 95 °C for 30 s; denaturation at 95 °C for 5 s, annealing at 58 °C for 30 s, extension at 60 °C for 34 s, 45 cycles. 2^−ΔΔCt^ method was used to analyze the data [42]. All experiments were performed with three technical replicates and three biological replicates, and the data were represented by mean value of three biological replicates.

### Prediction of target genes by CAMTA

Prediction of the target genes by CAMTA were performed as described by Yang and Poovaiah (2002) with some modifications [5]. 1-kb sequences upstream of the initiation codon (ATG) of all genes in wheat genome were collected as promoter sequences, and a search of *cis*-acting elements ACGCGG/CCGCGT (CGCG box) were conducted. The genes with more than two copies of CGCG box were considered as potential target genes by CAMTA. The MapMan tool was used to facilitate the assignment of different gene sets into functional categories (BINs). A MapMan mapping file that mapped the genes into BINs via hierarchical ontologies through the searching of a variety of reference databases was generated using the Mercator tool (http://mapman.gabipd.org/web/guest/app/mercator) [43].

## Supplementary information

**Additional file 1: Table S1.** Predicted target genes by CAMTA.

**Additional file 2: Table S2.** Primer sequences of *TaCAMTA* and *TaActin* genes used for qRT-PCR analysis.

## Data Availability

The data sets supporting the article are included within the article and its additional files.

## Abbreviations

CAMTA: Calmodulin-binding transcription activator;
CaM: Calmodulin
CML: Calmodulin-like proteins
CDPK: Calcium-dependent protein kinases
CBL: Calcineurin B-like proteins
CaMBP: Calmodulin-binding proteins
TIG: A transcription factor immunoglobulin-like DNA-binding domain
ANK: Ankyrin repeats
CaMBD: Calmodulin-binding domain
CBF2: C-repeat binding factor2
RD26: Responsive to dehydration26
ERD7: Early response to dehydration7
RAB18: Responsive to ABA18
LTPs: Lipid transfer proteins
COR78: Cold-regulated78
HSPs: Heat shock proteins
EDS1: Enhanced disease susceptibility1
SA: Salicylic acid
VIGS: Virus-induced gene silencing
NJ: Neighbor-joining
ABA: Abscisic acid
ABRE: ABA-responsive element
SARE: SA-responsive promoter element
P1BS: Phosphate starvation-responsive element
SURE: Sulfur-responsive element
DAA: Days after athesis
HMM: Hidden Markov Model
NCBI: National Center for Biotechnology Information
CDD: Conserved domain database
iTOL: Interactive Tree Of Life
GSDS: Gene Structure Display Server
qRT-PCR: Quantitative real-time PCR

## References

[1] Dodd AN, Kudla J, Sanders D (2010). The language of calcium signaling. Annu Rev Plant Biol.

[2] Hashimoto K, Kudla J (2011). Calcium decoding mechanisms in plants. Biochimie..

[3] Abbas N, Maurya JP, Senapati D, Gangappa SN, Chattopadhyay S (2014). Arabidopsis CAM7 and HY5 physically interact and directly bind to the *HY5* promoter to regulate its expression and thereby promote photomorphogenesis. Plant Cell.

[4] Wei M, Xu X, Li C (2017). Identification and expression of *CAMTA* genes in *Populus trichocarpa* under biotic and abiotic stress. Sci Rep.

[5] Yang T, Poovaiah BW (2002). A calmodulin-binding/CGCG box DNA-binding protein family involved in multiple signaling pathways in plants. J Biol Chem.

[6] Finkler A, Ashery-Padan R, Fromm H (2007). CAMTAs: calmodulin-binding transcription activators from plants to human. FEBS Lett.

[7] Bouche N, Scharlat A, Snedden W, Bouchez D, Fromm H (2002). A novel family of calmodulin-binding transcription activators in multicellular organisms. J Biol Chem.

[8] Yang TB, Peng H, Whitaker BD, Conway WS (2012). Characterization of a calcium/calmodulin regulated SR/CAMTA gene family during tomato fruit development and ripening. BMC Plant Biol.

[9] Choi MS, Kim MC, Yoo JH, Moon BC, Koo SC, Park BO (2005). Isolation of a calmodulin-binding transcription factor from rice (*Oryza sativa* L.). J Biol Chem.

[10] Shangguan L, Wang X, Leng X, Liu D, Ren G, Tao R (2014). Identification and bioinformatic analysis of signal responsive/calmodulin-binding transcription activators gene models in *Vitis vinifera*. Mol Biol Rep.

[11] Yue R, Lu C, Sun T, Peng T, Han X, Qi J (2015). Identification and expression profiling analysis of calmodulin-binding transcription activator genes in maize (Zea mays L.) under abiotic and biotic stresses. Front Plant Sci.

[12] Wang G, Zeng H, Hu X, Zhu Y, Chen Y, Shen C (2015). Identification and expression analyses of calmodulin-binding transcription activator genes in soybean. Plant Soil.

[13] Rahman H, Xu YP, Zhang XR, Cai XZ (2016). *Brassica napus* genome possesses extraordinary high number of *CAMTA* genes and *CAMTA3* contributes to PAMP triggered immunity and resistance to *Sclerotinia sclerotiorum*. Front Plant Sci.

[14] Yang Y, Sun T, Xu L, Pi E, Wang S, Wang H, Shen C (2015). Genome-wide identification of CAMTA gene family members in *Medicago truncatula* and their expression during root nodule symbiosis and hormone treatments. Front Plant Sci.

[15] Zhang J, Pan X, Ge T, Yi S, Lv Q, Zheng Y (2018). Genome-wide identification of citrus CAMTA genes and their expression analysis under stress and hormone treatments. J Hortic Sci Biotechnol.

[16] Kim Y, Park S, Gilmour SJ, Thomashow MF (2013). Roles of CAMTA transcription factors and salicylic acid in configuring the low-temperature transcriptome and freezing tolerance of Arabidopsis. Plant J.

[17] Doherty CJ, Van Buskirk HA, Myers SJ, Thomashow MF (2009). Roles for Arabidopsis CAMTA transcription factors in cold-regulated gene expression and freezing tolerance. Plant Cell.

[18] Pandey N, Ranjan A, Pant P, Tripathi RK, Ateek F, Pandey HP (2013). CAMTA 1 regulates drought responses in *Arabidopsis thaliana*. BMC Genomics.

[19] Du L, Ali GS, Simons KA, Hou J, Yang T, Reddy AS, Poovaiah BW (2009). Ca^(2+)^/calmodulin regulates salicylic-acid-mediated plant immunity. Nature..

[20] Wang Y, Wei F, Zhou H, Liu N, Niu X, Yan C (2019). TaCAMTA4, a calmodulin-interacting protein, involved in defense response of wheat to *Puccinia triticina*. Sci Rep.

[21] Pant P, Iqbal Z, Pandey BK, Sawant SV (2018). Genome-wide comparative and evolutionary analysis of calmodulin-binding transcription activator (CAMTA) family in *Gossypium* species. Sci Rep.

[22] Rahman H, Yang J, Xu YP, Munyampundu JP, Cai XZ (2016). Phylogeny of plant CAMTAs and role of AtCAMTAs in nonhost resistance to *Xanthomonas oryzae* pv *oryzae*. Front Plant Sci.

[23] Kaplan B, Davydov O, Knight H, Galon Y, Knight MR, Fluhr R, Fromm H (2006). Rapid transcriptome changes induced by cytosolic Ca^2+^ transients reveal ABRE-related sequences as Ca^2+^-responsive *cis* elements in Arabidopsis. Plant Cell.

[24] Després C, Chubak C, Rochon A, Clark R, Bethune T, Desveaux D, Fobert PR (2003). The Arabidopsis NPR1 disease resistance protein is a novel cofactor that confers redox regulation of DNA binding activity to the basic domain/leucine zipper transcription factor TGA1. Plant Cell.

[25] Chakravarthy S, Tuori RP, D'Ascenzo MD, Fobert PR, Despres C, Martin GB (2003). The tomato transcription factor Pti4 regulates defense-related gene expression via GCC box and non-GCC box cis elements. Plant Cell.

[26] Nishiuchi T, Shinshi H, Suzuki K (2004). Rapid and transient activation of transcription of the ERF3 gene by wounding in tobacco leaves: possible involvement of NtWRKYs and autorepression. J Biol Chem.

[27] Yu DQ, Chen CH, Chen ZX (2001). Evidence for an important role of WRKY DNA binding proteins in the regulation of *NPR1* gene expression. Plant Cell.

[28] Schunmann PH, Richardson AE, Smith FW, Delhaize E (2004). Characterization of promoter expression patterns derived from the *Pht1* phosphate transporter genes of barley (*Hordeum vulgare* L.). J Exp Bot.

[29] Maruyama-Nakashita A, Nakamura Y, Watanabe-Takahashi A, Inoue E, Yamaya T, Takahashi H (2005). Identification of a novel cis-acting element conferring sulfur deficiency response in Arabidopsis roots. Plant J.

[30] Li XH, Huang L, Zhang YF, Ouyang ZG, Hong YB, Zhang HJ (2014). Tomato SR/CAMTA transcription factors SlSR1 and SlSR3L negatively regulate disease resistance response and SlSR1L positively modulates drought stress tolerance. BMC Plant Biol.

[31] Buyuk I, Ilhan E, Sener D, Ozsoy AU, Aras S (2019). Genome-wide identification of *CAMTA* gene family members in *Phaseolus vulgaris* L. and their expression profiling during salt stress. Mol Biol Rep.

[32] Kersey PJ, Allen JE, Armean I, Boddu S, Bolt BJ, Carvalho-Silva D (2016). Ensembl genomes 2016: more genomes, more complexity. Nucleic Acids Res.

[33] Finn RD, Mistry J, Schuster-Bockler B, Griffiths-Jones S, Hollich V, Lassmann T (2006). Pfam: clans, web tools and services. Nucleic Acids Res.

[34] Wheeler TJ, Eddy SR (2013). Nhmmer: DNA homology search with profile HMMs. Bioinformatics..

[35] Chou KC, Shen HB (2010). Plant-mPLoc: a top-down strategy to augment the power for predicting plant protein subcellular localization. PLoS One.

[36] Kumar S, Stecher G, Li M, Knyaz C, Tamura K (2018). Molecular evolutionary genetics analysis across computing platforms. Mol Biol Evol.

[37] Letunic I, Bork P (2019). Interactive tree of life (iTOL) v4: recent updates and new developments. Nucleic Acids Res.

[38] Hu B, Jin J, Guo AY, Zhang H, Luo J, Gao G (2015). GSDS 2.0: an upgraded gene feature visualization server. Bioinformatics.

[39] Marchler-Bauer A, Bo Y, Han L, He J, Lanczycki CJ, Lu S (2017). CDD/SPARCLE: functional classification of proteins via subfamily domain architectures. Nucleic Acids Res.

[40] Higo K, Ugawa Y, Iwamoto M, Higo H (1998). PLACE: a database of plant cis-acting regulatory DNA elements. Nucleic Acids Res.

[41] Hoagland DR, Arnon DI (1950). The water culture method for growing plants without soil. Calif Agric Exp Sta Circ.

[42] Livak KJ, Schmittgen TD (2001). Analysis of relative gene expression data using real-time quantitative PCR and the 2^−ΔΔCT^ method. Methods..

[43] Thimm O, Bläsing O, Gibon Y, Nagel A, Meyer S, Krüger P, Selbig J, Müller LA, Rhee SY, Stitt M (2004). MAPMAN: a user-driven tool to display genomics datasets onto diagrams of metabolic pathways and other biological processes. Plant J.

